# Evaluation and practical application of prompt-driven ChatGPTs for EMR generation

**DOI:** 10.1038/s41746-025-01472-x

**Published:** 2025-02-02

**Authors:** Hanlin Ding, Wenjie Xia, Yujia Zhou, Lei Wei, Yipeng Feng, Zi Wang, Xuming Song, Rutao Li, Qixing Mao, Bing Chen, Hui Wang, Xing Huang, Bin Zhu, Dongyu Jiang, Jingyu Sun, Gaochao Dong, Feng Jiang

**Affiliations:** 1 Department of Thoracic Surgery, Nanjing Medical University Affiliated Cancer Hospital & Jiangsu Cancer Hospital & Jiangsu Institute of Cancer Research, 21009 Nanjing, China; 2 Jiangsu Key Laboratory of Molecular and Translational Cancer Research, Cancer Institute of Jiangsu Province, Nanjing, China; 3 The Fourth Clinical College of Nanjing Medical University, Nanjing, China; 4 The Second Clinical Medical School of Nanjing Medical University, Nanjing, China; 5 Department of Cardiothoracic Surgery, Jinling Hospital, Nanjing University School of Medicine, Nanjing, China; 6 Department of Thoracic Surgery, Dushu Lake Hospital Affiliated to Soochow University, Suzhou, China; 7 Pathological Department of Jiangsu Cancer Hospital, Nanjing, P. R. China; 8 Hospital Development Management Office, Nanjing Medical University, Nanjing, China; 9 Department of Orthopedics, Wuxi People’s Hospital Affiliated to Nanjing Medical University, Wuxi, China; 10 Department of Cardiology, First Affiliated Hospital of Nanjing Medical University, Jiangsu Province Hospital, Nanjing, China

**Keywords:** Population screening, Cancer screening

## Abstract

This study investigates the application of prompt engineering to optimize prompt-driven ChatGPT for generating electronic medical records (EMRs) during lung nodule screening. We assessed the performance of ChatGPT in generating EMRs from patient–provider verbal consultations and integrated this approach into practical tools, such as WeChat mini-programs, accessible to patients before hospital visits. The findings highlight ChatGPT’s potential to enhance workflow efficiency and improve diagnostic processes in clinical settings.

## Introduction

The volume of digital healthcare data has grown exponentially over the past two decades^1^. Electronic medical records (EMRs) have become indispensable tools for clinicians, serving as repositories of clinical information; early symptom indicators, and treatment histories^2^. Well-structured EMRs can provide valuable insights into risk factors and early disease markers, aiding in preemptive healthcare strategies^3,4^.

However, generating EMRs requires comprehensive physical examinations and meticulous patient medical history collection—processes that are time-intensive and prone to errors^5,6^. Although EMRs improve risk assessment and decision-making^7,8^, variability in documentation practices and disparities in clinical experience can compromise their utility^9^. Moreover, many clinicians report that EMR systems are cumbersome and difficult to navigate^10,11^, resulting in significant time spent reviewing records^12^. These inefficiencies, combined with outdated regulatory frameworks and limited healthcare system priorities, contribute to clinician fatigue^13–15^ and reduced productivity^16,17^.

Addressing these challenges requires the development of intuitive EMR interfaces and advanced data management strategies in clinical decision-making and patient care. A technological solution capable of streamlining the collection of patient history during the initial phases of diagnosis could enhance the efficiency and accuracy of medical documentation. By automating foundational tasks, healthcare providers can focus on delivering more effective and personalized care and reducing documentation errors, ultimately fostering better therapeutic outcomes^18,19^.

Large language models (LLMs), exemplified by OpenAI’s ChatGPT, have shown promise in clinical settings due to their ability to emulate human dialogue and process textual information^20–24^. Studies have demonstrated ChatGPT’s capacity to identify high-risk patients through EMRs or other medical records (e.g., from the NEJM clinicopathologic conferences)^25,26^, process medical records, respond to patient inquiries, and support clinical diagnosis^27,28^. As the availability and accessibility of ChatGPT have increased, establishing the uses and limitations of LLMs used in clinical practice is critical. However, the consistency and accuracy of LLM-generated outputs in response to structured clinical tasks remain areas of active research^29^. Prompt engineering, a technique used to precisely guide LLMs in generating context-specific and accurate responses, offers a potential solution to these limitations^30–32^. By designing precise and task-oriented prompts, the reliability of LLM outputs can be significantly enhanced^29–31^. Research has already highlighted LLMs’ capabilities in extracting valuable insights from text and their effectiveness in various clinical applications, such as analyzing computed tomography and pathology reports and generating nursing care plans.

Lung cancer, a leading cause of cancer-related mortality worldwide^33^, serves as an ideal case study for exploring the application of LLMs in EMR generation. Early screening, diagnosis, and intervention can effectively reduce the occurrence of advanced-stage lung cancer, thereby improving patient outcomes. Despite the demonstrated potential of LLMs in processing EMR data, including computed tomography and pathology reports, and formulating nursing care plans^29,34^, their ability to extract information from medical consultations and create EMRs remains underexplored.

**Fig. 1:**
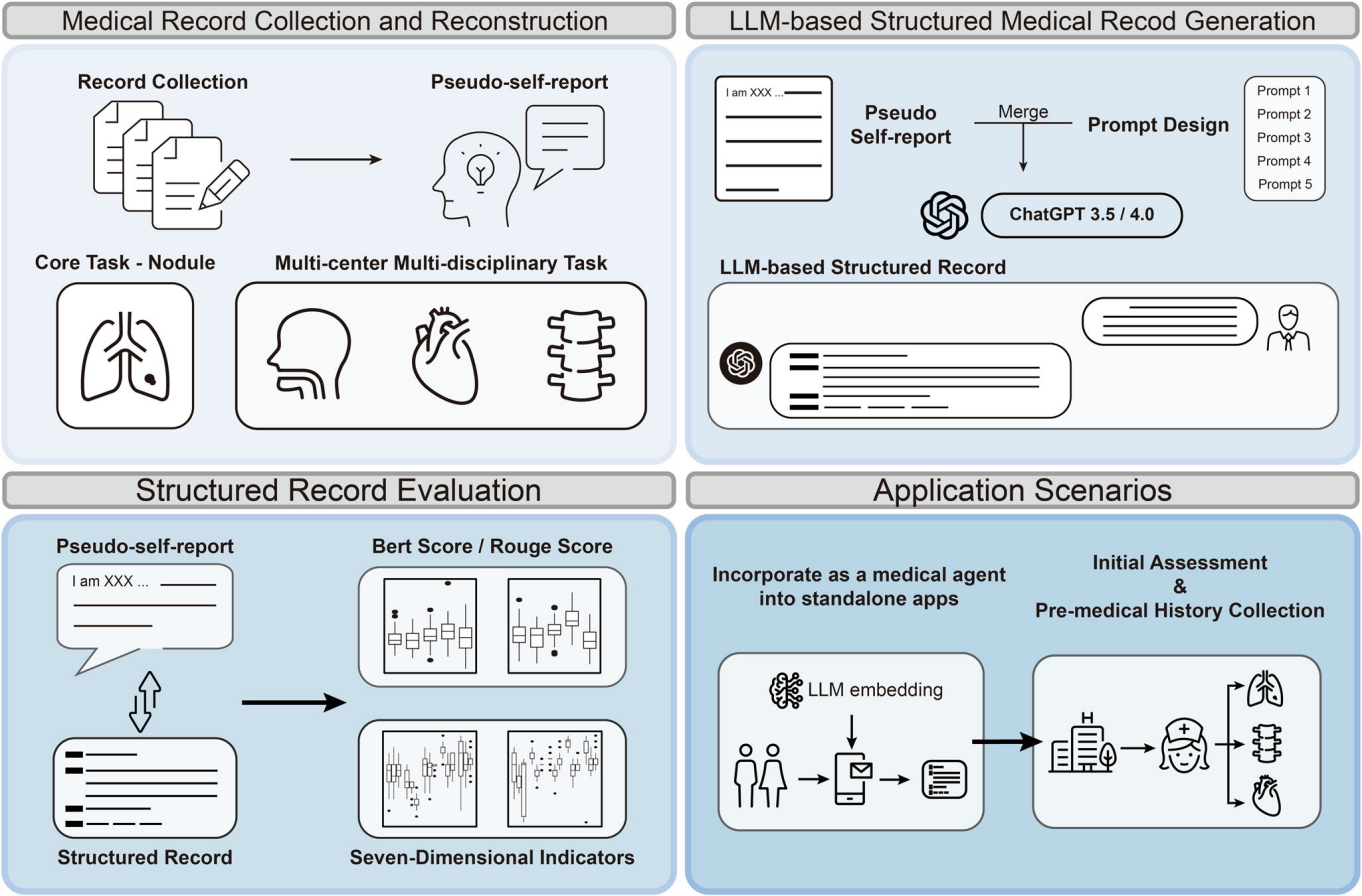
An overview of the study. Multidisciplinary EMRs were collected and converted into a pseudo-self-report format. The pseudo-reports were sent to the ChatGPT by combining with five delicately designed prompts. Finally, the generated EMRs were evaluated, and the potential application scenarios were constructed.

In this study, we focused on lung cancer as a case study to evaluate the feasibility of using LLMs for EMR generation. We developed prompt-based methodologies to standardize EMR creation from initial medical consultations and assessed the performance of ChatGPT-3.5 and GPT-4 using various prompt designs. Furthermore, we proposed a practical application scenario incorporating these prompts into a real-world tool for clinical use. Figure 1 outlines the study’s workflow.

## Materials and methods

### Study design

This study was approved by the Institutional Review Board of the Affiliated Cancer Hospital of Nanjing Medical University (KY-2024-091). As illustrated in Fig. 1, the study was conducted in the following four stages: (i) multidisciplinary EMRs were collected from three medical centers and the pseudo-self-report of the patients were converted; (ii) five prompts were designed and structured medical records were generated through ChatGPT models; (iii) the performance of the prompt-driven ChatGPT was evaluated using three metrics; (iv) the potential application scenarios were delineated.

### Medical record acquisition and conversion

The medical records of the patients with lung nodules at the Affiliated Cancer Hospital of Nanjing Medical University were comprehensively collected as the initial assessment materials for the Prompt task. This study included the EMRs of esophageal cancer, which is a major thoracic surgery condition using two external datasets from nonthoracic surgery specialties: cardiology cases from Jiangsu Provincial People’s Hospital and orthopedic cases from The Affiliated Wuxi People’s Hospital of Nanjing Medical University for the evaluation of the generalizability of the Prompt task.

As depicted in Supplementary Fig. 1, 103 EMRs were included in the study. Among these, the data on 48 (46.6%) lung nodule cases and 20 (19.4%) esophageal cancer cases were collected from the Affiliated Cancer Hospital of Nanjing Medical University. In addition, the data on 5 cardiology cases were collected from the First Affiliated Hospital of Nanjing Medical University and 30 (29.1%) orthopedic cases from The Affiliated Wuxi People’s Hospital of Nanjing Medical University to evaluate the generalization.

All EMRs were converted in the form of medical history reported by patients and subjected to basic quality control by professional clinicians (W.J.X., Q.X.M., B.C., and H.W.). The quality control standards were as follows: (1) complete past medical history; (2) clear admission and discharge diagnoses; (3) the content of the pseudo-self-report essentially including all key points of the medical records; (4) pseudo-self-report consistent with the tone and content of a real patient in medical consultation. Moreover, personal information (such as name, age, sex, and address) was randomly modified during the conversion process to protect patient privacy.

### LLM model

Our study employed two versions of ChatGPT (version GPT-3.5 and GPT-4; OpenAI, CA, USA) between January 18 and June 29, 2023. The GPT-4 model encompasses more parameters and computational power than its predecessor GPT-3.5^35^. We incorporated both GPT-3.5 and GPT-4 into our evaluation. The two ChatGPT models used in this study were integrated into the poe.com platform The parameters were set to default and the temperature was set to 0.3.

### Prompt design

Following the principles of “Best Practices For Text Generation Prompts” available on the poe.com platform, we designed five sets of prompts ranging in complexity. These prompts were tailored to standardize LLM outputs and enhance the accuracy and consistency of EMRs.

The five sets of prompts were developed iteratively, evolving from simple instructions to more complex and structured designs. Prompt 1 was a baseline prompt built using the Zero-shot prompting method. It used a simple task description to elicit EMR generation without additional context or examples. Prompts 2 and 3 applied the few-shot prompting technique. They introduced contextual information to guide the model. Prompt 2 defined the professional identity of the responder, while Prompt 3 provided simple task descriptions to further refine outputs. Prompt 4 further detailed the individual components of an EMR and broke down the generation task into structured steps, constructing a simple Chain of Thought (CoT), a logical sequence, to ensure comprehensive outputs. Leveraging the CRISPE framework, Prompt 5 introduced a highly detailed and standardized approach to EMR generation. The prompt structured the task into discrete sections, aligning closely with professional documentation standards. Supplementary Note 1 presents the detailed descriptions of the prompt design.

All prompts were meticulously integrated with pseudo-self-reported data, which were then input into GPT for EMR generation.

### Assessment of LLM-generated EMRs using different prompts

To assess the performance of ChatGPT across various prompt conditions, we employed both quantitative and qualitative evaluation methods. The BERTscore and Recall-Oriented Understudy for Gisting Evaluation (ROUGE) metrics were used to evaluate the summarization accuracy and relevance of the generated EMRs. These metrics provided insights into the linguistic quality and fidelity of the outputs^36,37^. Concurrently, given the high professional specificity of EMRs, clinical experts evaluated the outputs for critical attributes such as the quality of the chief complaint and the history of present illness. Building on previous studies, which reported four dimensions of “Key Result,” “Coherence,” “Usefulness,” and “Readability,” this study further expanded the assessment framework to include seven dimensions to evaluate the performance of the model (seven-dimensional indicators, 7DI)^38^. Detailed evaluation criteria are presented in Supplementary Note 2. Three experienced physicians (W.J.X., Q.X.M., and B.C.) reviewed and scored the generated EMRs for pulmonary nodules, while multidisciplinary EMRs were assessed by two experienced physicians from the corresponding departments.

### Construction of potential application scenarios

To demonstrate real-world applicability, we developed two systems utilizing the advanced prompts: AutoMedicalAgent-CN and an automated medical record system. Using Prompt-5, we created AutoMedicalAgent-CN, an AI-driven medical assistant on the Poe.com platform (Quora, Inc., https://poe.com/) powered by the chatbot GPT-3.5-Turbo. This chatbot conducted patient interactions through six guided questions and collected input data. This input data was the obtained information to generate the corresponding medical record text.

A second system, based on Prompt-4, integrated questionnaires and voice recognition technologies. Five to six relevant preset questions collected patient information. Subsequently, Text-to-Speech APIs (Baidu, Inc.) converted questions into audio for patient engagement, and Speech Recognition APIs (Baidu, Inc.) transcribed spoken responses into text. Ultimately, the transcribed content, combined with the Prompt-4 text, was processed by ChatGPT to produce structured medical records. The entire system was implemented using Python and Baidu’s Python APIs to ensure seamless integration of voice and text-based technologies.

To evaluate the integration potential of the automated medical record generation system into clinical settings, we collected voice recordings of Q&A sessions with 10 patients. These were transcribed into pseudo-self-reports, which were then used to generate EMRs through the Prompt-4-based system. The generated EMRs were compared using the 7DI scoring system to determine the system’s effectiveness when processing Q&A voice recordings. This evaluation aimed to assess whether the Prompt-4-based system could be effectively incorporated into real clinical environments.

To further evaluate the system’s contribution to enhancing clinical efficiency, we measured the time required for two clinicians to consult 100 patients in a real-world setting. Two experienced clinicians—one senior (W.J.X.) and one junior (B.C.)—conducted medical consultations independently for 100 randomly selected patients, and the time spent on each consultation was recorded. Additionally, we documented the time required for consultations involving another 100 patients, where the two clinicians had access to pre-generated EMRs from the Prompt-4-based system. This comparison aimed to validate the system’s translational value in reducing consultation time and improving workflow efficiency.

### Statistical analysis

Text segmentation was performed using the Python-based Natural Language Toolkit (NLTK) and Jieba libraries^39^. The Bert-score library was used to compute BERT scores, while the ROUGE library^40^ was employed to calculate ROUGE-1, ROUGE-2, and ROUGE-L metrics. Statistical results were presented as mean ± standard deviation. To analyze differences in performance across two versions of ChatGPT and among different prompts, T-tests were conducted using the SciPy library^41^. Statistical significance was set at *P* < 0.05, with all tests being two-sided. Data processing, statistical analysis, and visualization were conducted using Python (version 3.7) and R (version 4.0.5) software.

## Results

### Core task: automated generation of pulmonary nodule EMRs

**Fig. 2:**
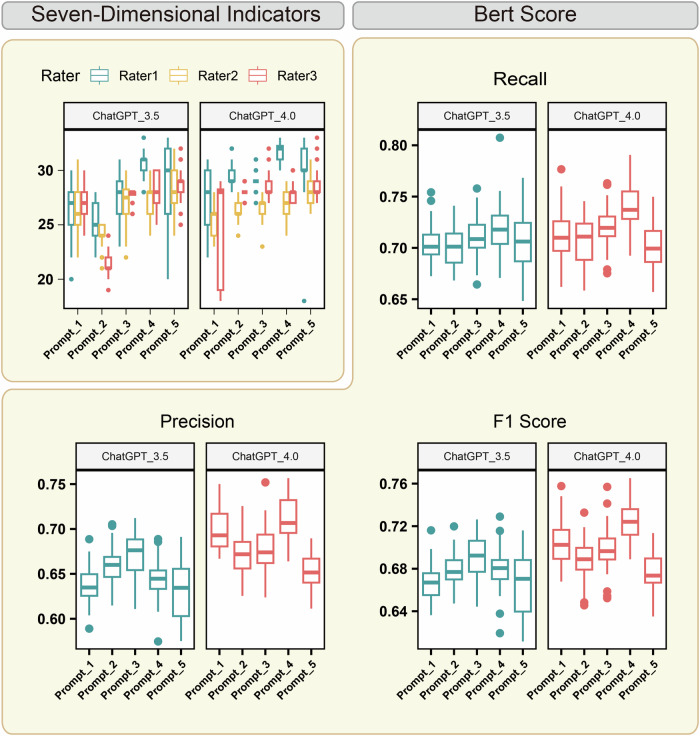
Evaluation of the lung nodule EMRs created by LLMs. The total score of 7DI (top-left panel) and the BERTscore in evaluating lung nodule EMRs created by prompt-driven LLMs.

The study focused on generating EMRs for patients with pulmonary nodules based on pseudo-self-reports. Results demonstrated that the GPT-4 model outperformed GPT-3.5 in both accuracy and consistency. Specifically, Prompt-4 achieved the highest BERT scores for recall (0.74 ± 0.0), precision (0.71 ± 0.02), and F1-score (0.73 ± 0.02) (Fig. 2). For the ROUGE metrics, Prompt-4 performed best in ROUGE-2 scoring (0.13 ± 0.05), whereas Prompt-3 achieved the highest recall for ROUGE-1 (0.38 ± 0.08) and ROUGE-L (0.26 ± 0.07) scores (Supplementary Fig. 2). GPT-4 consistently outperformed GPT-3.5 across all BERT and ROUGE metrics under various prompt conditions. Notably, Prompt-1 and Prompt-4 produced statistically significant differences in results between the two models. While GPT-4 demonstrated superior F1 scores, precision differences between the two models were minimal (Supplementary Table 1).

The quality of the EMRs was further evaluated using the 7DI scoring system by experienced physicians (Supplementary Table 2, Fig. 2). GPT-4-generated texts received higher overall evaluations, achieving a mean 7DI score of 28.20 ± 2.36. Texts generated with Prompt-3, Prompt-4, and Prompt-5 under the GPT-4 model were consistently rated as high quality by all three expert raters. Rater 1 rated Prompt-4-generated texts the highest (31.69 ± 1.03). Raters 2 and 3 preferred texts generated by Prompt-5, assigning identical scores (Raters 2: 28.69 ± 1.40; Rater 3: 29.06 ± 1.37).

### Analysis of generalizability

To evaluate the generalizability of LLMs diverse medical contexts, we assessed their ability to generate EMRs for multidisciplinary cases, including esophageal cancer, cardiology, and orthopedics, using data from multiple centers^42^.

**Fig. 3:**
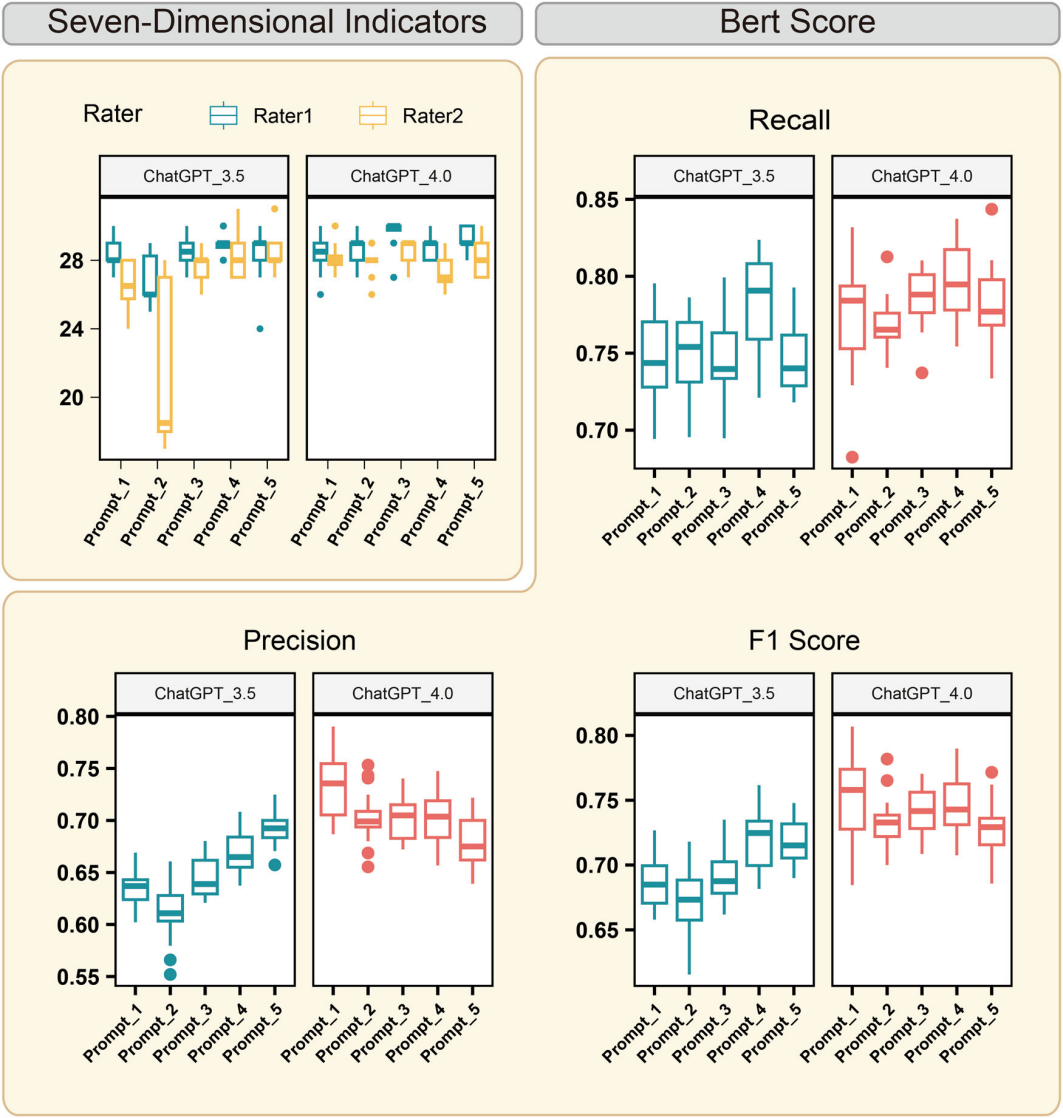
Evaluation of the esophageal cancer EMRs created by LLMs. The total score of 7DI (top-left panel) and the BERTscore in evaluating esophageal cancer EMRs created by prompt-driven LLMs.

We first compared the BERT and ROUGE scores for EMR generation related to esophageal cancer (Fig. 3, Supplementary Fig. 3). Across all prompt sets, GPT-4 consistently outperformed GPT-3.5, achieving higher recall rates. Using Prompt-4, GPT-4 achieved the highest BERTscore recall (0.80 ± 0.03) and ROUGE-2 recall (0.25 ± 0.09). Additionally, it demonstrated strong performance in ROUGE-1 (0.44 ± 0.09) and ROUGE-L (0.32 ± 0.06), though these scores were slightly surpassed by Prompt-1. The F1 scores followed a similar trend, highlighting GPT-4’s superior ability to extract accurate information from pseudo-self-reports (Supplementary Table 3). Clinical evaluations using the 7DI further validated these findings. Prompt-3 under GPT-4 achieved the highest 7DI score of 29.02 ± 1.00 (Fig. 3, Supplementary Fig. 3, Supplementary Table 4), showcasing its optimal performance for esophageal cancer EMR generation.

**Fig. 4:**
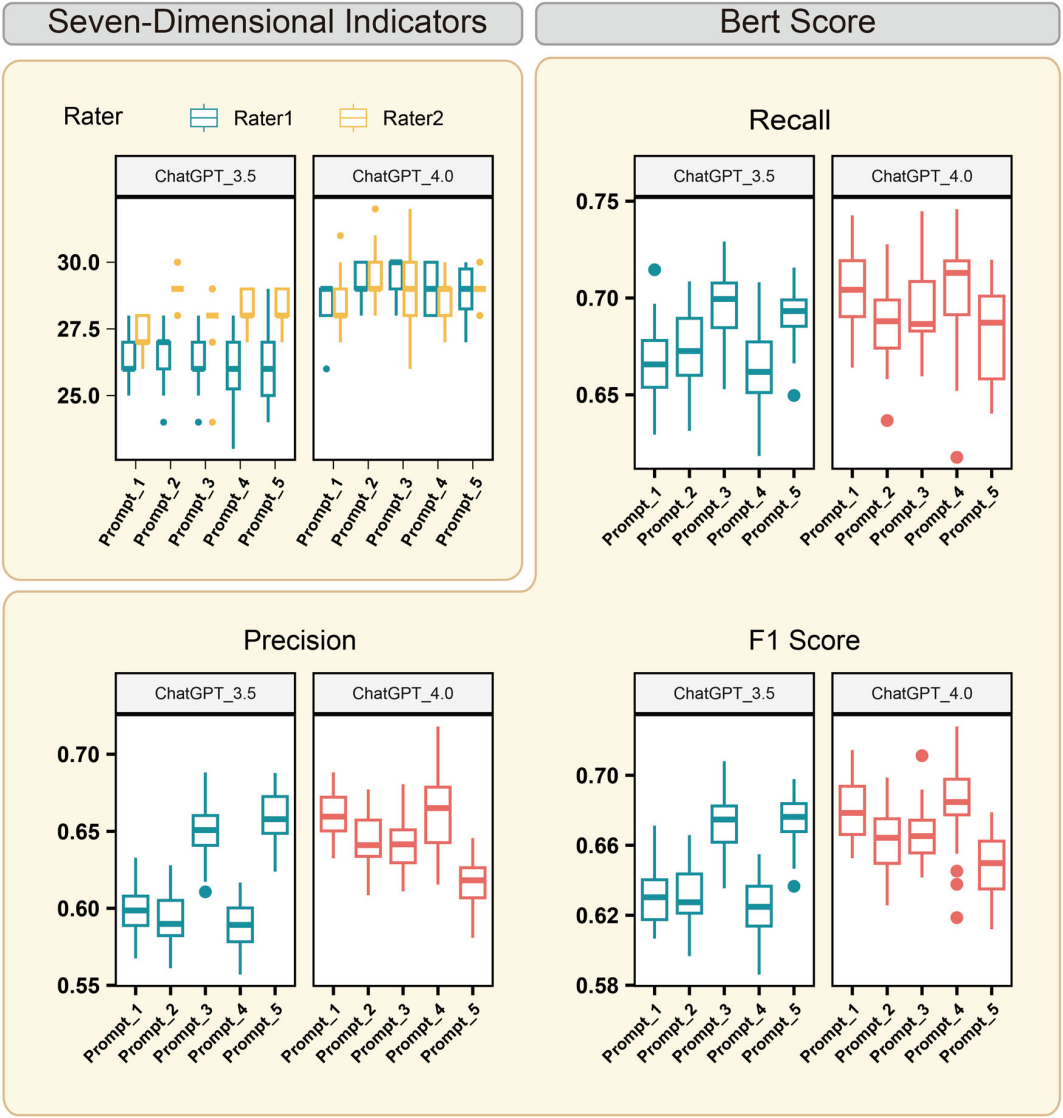
Evaluation of the orthopedic EMRs created by LLMs. A total score of 7DI (top-left panel) and the BERTscore in evaluating orthopedic EMRs created by prompt-driven LLMs.

In orthopedic cases, the Prompt-4-driven GPT-4 model outperformed other configurations, achieving a BERTscore recall of 0.71 ± 0.03, precision of 0.66 ± 0.03, and an F1-score of 0.68 ± 0.02. Its ROUGE scores also aligned with the previously observed trends, further reinforcing its effectiveness (Fig. 4, Supplementary Fig. 4). The 7DI evaluations similarly favored the Prompt-4-driven GPT-4 model, as evaluators rated its tone and content highly (Supplementary Tables 5, 6).

In the cardiology dataset (Supplementary Figs. 5 and 6), GPT-4 demonstrated superior recall rates for EMR generation, though the differences in BERTscore recall between GPT-3.5 and GPT-4 for Prompt-3 were not statistically significant, likely due to the limited sample size. Despite moderate performance in BERTscore and ROUGE metrics, clinical experts evaluated Prompt-4 and Prompt-5 favorably, further attesting to their usability in cardiology-related EMR generation (Supplementary Tables 7 and 8).

### Transition scenes can help clinicians enhance efficiency

To demonstrate the practical application of LLM-driven EMRs, we developed two systems based on Prompt-4 and Prompt-5 to streamline medical record generation: Chatbot for Automated EMR Generation: Using Prompt-5, we built a chatbot capable of generating EMRs through interactive Q&A sessions (Supplementary Fig. 7).

**Fig. 5:**
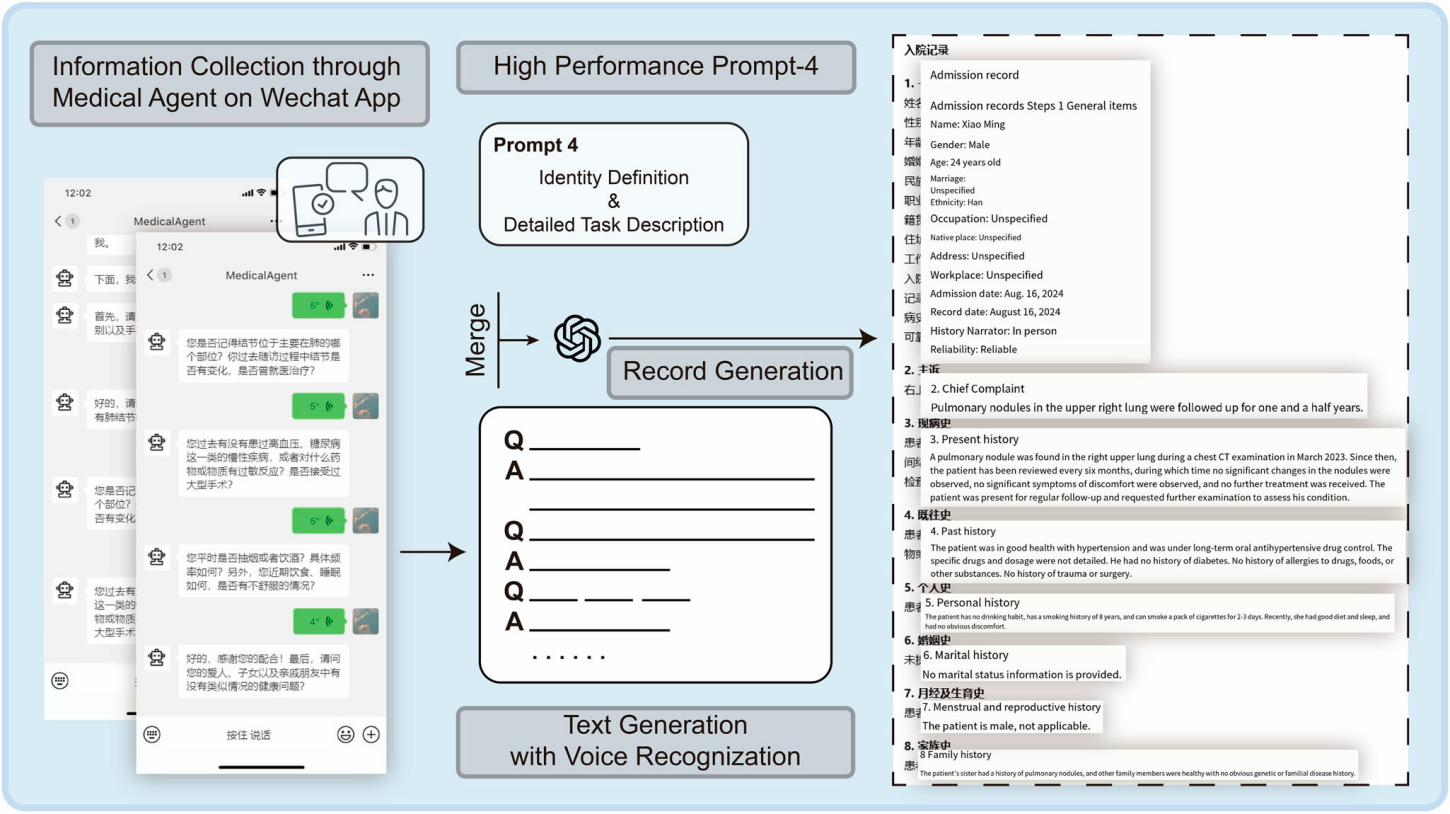
Construction of translational scenarios. In the workflow of the automated medical record-generation system, several preset questions were asked and recorded through voice recognition technology. Then, by combining with prompt 4, the text was sent to the LLM for further EMR generation.

Voice Recognition Integrated Workflow: Leveraging Prompt-4, we constructed an automated system incorporating voice recognition for pre-collecting medical history before hospital visits. This workflow involved broadcasting preset questions, transcribing patient responses via Baidu’s Speech Recognition API, and integrating the collected data into ChatGPT for EMR generation (Supplementary Movie S1 and Fig. 5).

To validate the clinical relevance of these systems, we generated EMRs from 10 patient Q&A voice recordings and their corresponding pseudo-self-reports (Supplementary Table 9). The 7DI scores for EMRs from voice records (29.90 ± 1.20) and pseudo-self-reports (29.20 ± 1.14) showed no significant differences, indicating the robustness of the system. However, clinician-authored EMRs scored higher overall (31.40 ± 1.17), particularly in the Chief Complaint category, where scores for LLM-generated EMRs were notably lower (3.30 ± 0.48 and 3.50 ± 0.53 for pseudo-self-reports and voice records, respectively) (Supplementary Table 9).

We further assessed the impact of LLM-generated EMRs on consultation efficiency by documenting the time required for two clinicians to consult with 100 patients, with and without the aid of pre-generated EMRs (Supplementary Fig. 8). The results demonstrated a significant reduction in consultation time for junior clinicians when using the automated system, decreasing from 9.56 ± 4.97 min to 7.41 ± 4.74 min (*P* < 0.005) (Supplementary Table 9). Furthermore, with LLM-generated EMRs, junior physicians achieved efficiency comparable to senior physicians (9.56 ± 4.97 min vs 7.65 ± 4.19 min, *P* < 0.005). These findings underscore the clinical value of prompt-driven LLMs in enhancing consultation workflows, particularly in initial nodule screening and patient history collection (Supplementary Table 10).

## Discussion

Timely screening and early detection are crucial to reducing lung cancer mortality rates^43,44^. However, existing research has predominantly focused on disease detection and risk assessment, with limited efforts devoted to optimizing the screening process itself. Primary screening often faces challenges such as resource constraints, time limitations, and inefficient post-screening management pathways^45^. Furthermore, the growing reliance on EMRs, compounded by their poor usability, high volumes of patient messages, and time-consuming data entry requirements, contributes to clinician fatigue and decreased efficiency^46–50^. To address these issues, initiatives that assist clinicians in structuring EMRs and extracting critical information can enhance diagnostic efficiency and alleviate clinician workload.

This study explored the application of prompt-driven LLMs for automating the generation of structured EMRs from patients’ medical consultation data, to improve screening efficiency and facilitate clinical decision-making. Our study demonstrated a significant enhancement in the performance of GPT-4 compared with GPT-3.5 across metrics such as BERTscore, ROUGE, and 7DI. Notably, the quality of the generated text improved as the complexity of prompts increased, consistent with prior research on prompt engineering^30,51^. Moreover, refined prompt designs enabled GPT-3.5 to match the performance of GPT-4, underscoring the critical role of optimized prompts in maximizing LLM functionality. Furthermore, in a multidisciplinary medical record context, we observed that prompts tailored for a specific discipline retained their effectiveness even when applied to broader frameworks. This adaptability highlights the potential of prompt-driven LLMs in various clinical scenarios.

To harness the capabilities of prompt-driven LLMs in clinical settings, we developed two practical applications: Prompt-4 for automated EMR generation and Prompt-5 for interactive diagnostics. These tools were designed to integrate seamlessly into the clinical workflow, thus improving efficiency and precision in patient care.

For Prompt-5, this scenario enabled LLMs to collect patient information through a structured question-and-answer format. A Prompt-5-based automated medical agent was developed on the poe.com platform, offering an interactive diagnostic experience for patients and clinicians. For Prompt-4, integrated with voice recognition and playback, this system collected patient information through preset questions and used a Prompt-4-driven LLM to generate preliminary EMRs prior to hospital visits. This tool was deployed as a medical agent within WeChat Mini Programs and standalone apps, providing clinicians with valuable references to enhance diagnostic efficiency. This innovative approach streamlined the workflow and augmented the precision of diagnostic procedures, offering a valuable tool to medical professionals that provides a seamless patient experience. Real-world testing revealed a notable improvement in the efficiency of junior clinicians when using EMRs generated by the Prompt-4 system.

Despite its innovative contributions, this study faced several limitations. The evaluation involved 103 multidisciplinary cases, which, while sufficient for demonstrating feasibility, requires validation on larger datasets and across more specialties to establish broader applicability. While prompts designed for pulmonary nodules exhibited broad utility, further customization is necessary for specific clinical scenarios to optimize model performance^52^. The synthesis of the “Chief Complaint” section often scored lower on 7DI, indicating room for improvement in aligning artificial intelligence-generated outputs with clinical documentation standards. Furthermore, the Prompt-4-based EMR leveraged OpenAI’s ChatGPT platform, raising potential concerns about data privacy and security. Although the study received approval from the Institutional Review Board of Jiangsu Cancer Hospital and the Jiangsu Institute of Cancer Research, future implementations must prioritize robust safeguards to protect patient data and mitigate legal risks. Finally, given the limitations of the native language proficiency of researchers, this study primarily focused on Mandarin Chinese scenarios. Extending validation to multilingual contexts will be essential for broader adoption.

Our study unveiled the potential of prompt-driven LLMs as valuable tools to support clinicians in diagnostic processes, particularly for lung nodule screening. These tools streamline critical processes such as initial patient screening and large-scale patient screening, improving communication during patient visits and enhancing diagnostic accuracy. The integration of such technologies can revolutionize patient–doctor interactions, optimize outpatient workflows, and elevate the overall quality of care. By augmenting physician efficiency and diagnostic accuracy, these innovations hold the promise of transforming clinical practice and improving patient outcomes.

## Supplementary information

Supplementary Information

Supplementary Movie 1

## Data Availability

The data that support the findings of this study are available from the corresponding author upon reasonable request.

## Code availability

Software code generated to support primary data analysis is available via the link below (https://github.com/dicklim/AutoMedicalGeneration-Demo).
